# Study on the rationality of small diameter metallic airway stent in treatment of tracheal stenosis in injured rabbits

**DOI:** 10.1186/s13019-023-02470-4

**Published:** 2024-03-05

**Authors:** Xiaoxiao Li, Changguo Wang, Ziyi Liu, Kai Wu, Zhenyu Yang, Daxiong Zeng, Dang Lin, Junhong Jiang

**Affiliations:** 1 Department of Respiratory and Critical Care Medicine, The First Affiliated Hospital of Soochow University, Suzhou, Jiangsu 215000 China; 2 Department of Respiratory and Critical Care Medicine, Dushu Lake Hospital to Soochow University, Suzhou, Jiangsu 215000 China; 3 Department of Respiratory and Critical Care Medicine, Suzhou Municipal Hospital (Eastern District), Suzhou, Jiangsu 215000 China

**Keywords:** Benign airway stenosis, Stent diameter selection, Complications, IL-8, MMP9

## Abstract

**Background:**

To observe the occurrence of related complications after self-expandable metallic (SEM) airway stents implantation with different diameters at different time points, and to provide theoretical basis for the optimal chioce of existing airway stents in clinical practice.

**Methods:**

Healthy New Zealand white rabbits were used to establish benign tracheal stenosis models after chest CT examination. Forty-fivemodel rabbits with more than 50% of airway stenosis were divided into two groups. Small-diameter SEM stents (The ratio of stent diameter to airway diameter is nearly 1.0) were implanted in Group A in 21 rabbits, and large-diameter tracheal stents (The ratio of stent diameter to airway diameter is more than 1.2) were implanted in Group B in 24 rabbits. Stent-related complications were observed after stent implantation in 2nd,4th,8th, and 12th week by bronchoscopygross anatomy, pathological and the expressions of IL-1RA, IL-8 and MMP9 in involved tracheal.

**Results:**

The incidence rate of tracheomalacia of stent was significantly higher in group B (24/24 100%) than that in group A (1 /21,4.8%) (*P* < 0.05). The incidence rate of scar contracture at both ends of stent was significantly higher than in group B (11 / 24,45.8%) that in group A (2 /21, 9.5%) (*P* < 0.05). The pathological results of both A and B showed that the columnar epithelium of bronchial mucosa began to damage and detach, inflammatory cells infiltrated after 2nd and 4th week of stenting, The epithelium was repaired, the lamina propria glands almost disappeared, collagen fiber proliferation was obvious, and scars were formed after 8th and 12th week of stenting. ELISA results revealed that the expressions of IL-1RA, IL-8, and MMP9 were increased in the stent group than in model rabbit with benign tracheal stenosis. IL-1RA and MMP9 increased at different periods in group B, but the expression of IL-1RA and MMP9 showed a tread of increasing in the early stage and then decreasing in group A.

**Conclusion:**

Metal stents can cause different degrees of stent-related complications in rabbits with benign tracheal stenosis. The incidence of stent-induced tracheomalacia and scar contracture were higher in Group B than that in Group A. IL-1RA, IL-8 and MMP9 may be involved in the development of complications after stentimplantation and peak value of group B movered backward. ing.

## Introduction

Benign airway stenosis is caused by various non-malignant tumor lesions, the most common of which include endotracheal intubation, stenosis after tracheotomy, tuberculosis, endobronchial benign tumors and tracheobronchomalacia. Benign airway stenosis is mainly treated by surgery and airway intervention treatment, and its development is limited due to the high risk and difficulty of surgical treatment. With the development of interventional pulmonology, airway stenting has become the first choice for the treatment of complex airway stenosis that is not suitable for surgical resection, and its short-term overall success rate can reach 87% [1], which can quickly -open the airway and improve the symptoms of dyspnea.

However, airway stenrelated complications limited in the use of benign airway stenosis, the common complications are granulation formation, scar contracture, secretion retention [2–4], Many researchers have reported granulation formation maybe relate with the stent size [5–7]. large size stent will increase the compression on the tracheal wall and aggravate the tracheal injury [8]. Carrau [9]noted granulation formation also was relation to the stent expansion force. However, it is inconclusive how to select the appropriate tracheal stent diameter in order to minimize the stent-ralted complications. Dr Hu [10] compared different diameters of Dumon stents on granulation tissue proliferation, and the results showed that when the stent-to-tracheal diameter ratio was > 100%, 90 − 100%, and < 90%, respectively, the granulation tissue proliferation rates were 42.86%, 26.32%, and 0%, respectively. However, whether the conclusion is sutable for the metal stent made by Nanjing Micro-Technology Company China There are no reported. So We selectedmetal stents produced by Nanjing Micro-Technology Company, PR China. to implanttwo different diameters of tracheal stentsin rabbits with benign tracheal stenosis. and to observe the incidence of stent-related complications So as to provide a theoretical basis to chooseoptimal diameter tracheal metal stents.

## Methods

### Establishment of rabbit model of benign tracheal stenosis

The 4-month-old white New Zealand rabbits with weight of 3.2 -3.5 kg were purchased and raised in Huqiao Biotechnology Co., Ltd. (license number: SYXK (Su) 2015-0002). Rabbits were kept in cages with free access to food in an animal room with a relative humidity of 40-50%, temperature of 22–25℃, and 12 h/12 h light and dark alternates. The Animal Experimental Ethics Committee approved the experimental protocol and surgical procedures of Soochow University (No.: 2018 − 185).

The experimental rabbits were anesthetized by rapid intramuscular injection of Xylazine hydrochloride injection 0.1ml/kg in the buttocks before surgery. CT scanning was performed before the test. The fat window (window width 5HU, window position − 100HU) was used to measure the diameter of rabbits. The diameter is measured as (H + Z)/2. Where H is the maximum transverse diameter of the tracheal lumen at the narrowest point and Z is the longitudinal diameter of the tracheal lumen perpendicular to H. The mean tracheal diameter of the rabbits was determined to be 5.97 ± 0.33 mm.

The airway mucosa and cartilage were damaged by the modeling method of Nakagishi [11] in 60/100 experimental rabbits. They were treated with 30 U of long-acting penicillin intramuscularly and daily oral SMZ to prevent acute upper respiratory tract infection after surgery,.

**Fig. 1:**
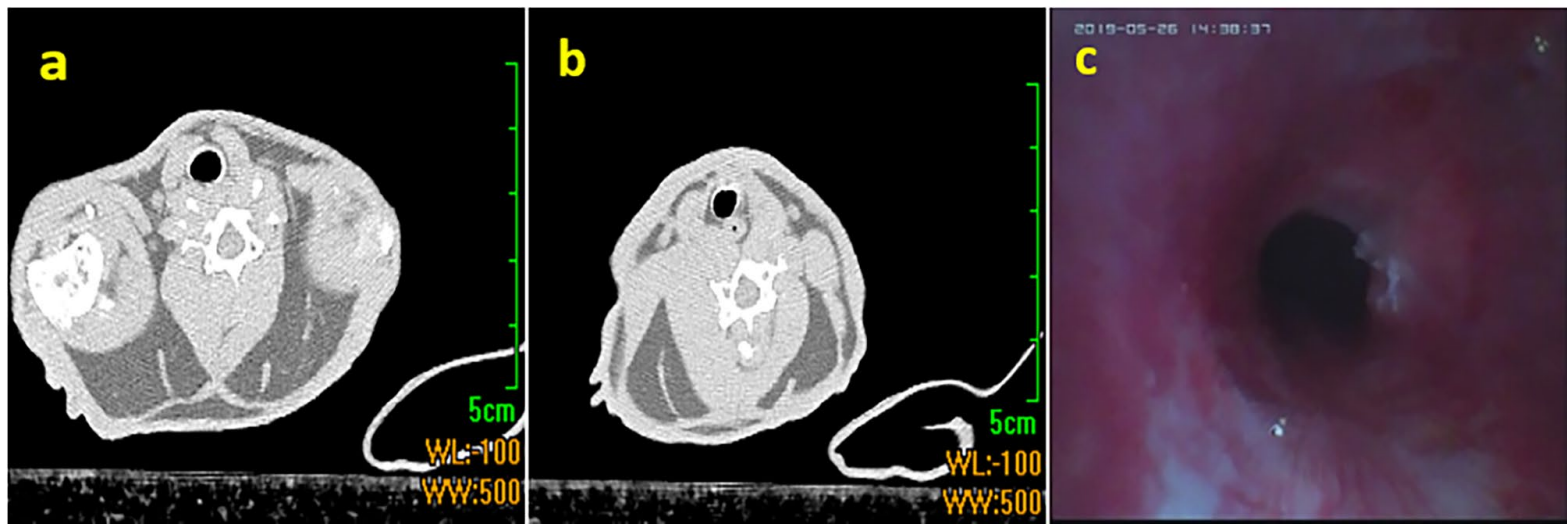
Different types of mediastinal windows of Rabbit trachea CT-scan **a**: normal trachea; **b**: stenotic trachea; **c**: successful modeling

Tracheal stenosis (Fig. 1a,b) was confirmed by Chest CT reexamination after 1 month of modeling. Success rate of modelling was 60% (/100) Forty-five rabbits with more than 50% airway stenosis (Fig. 1c) were randomly divided into two groups. In group A, Small-diameter tracheal stents (The ratio of stent diameter to airway diameter is nearly 1.0) were implanted in 21 experimental rabbits. In group B, large-diameter tracheal stents (ratio of stent diameter to tracheal transverse diameteris over1.2) were implanted in 24 experimental rabbits.

### Stent and delivery system

**Fig. 2:**
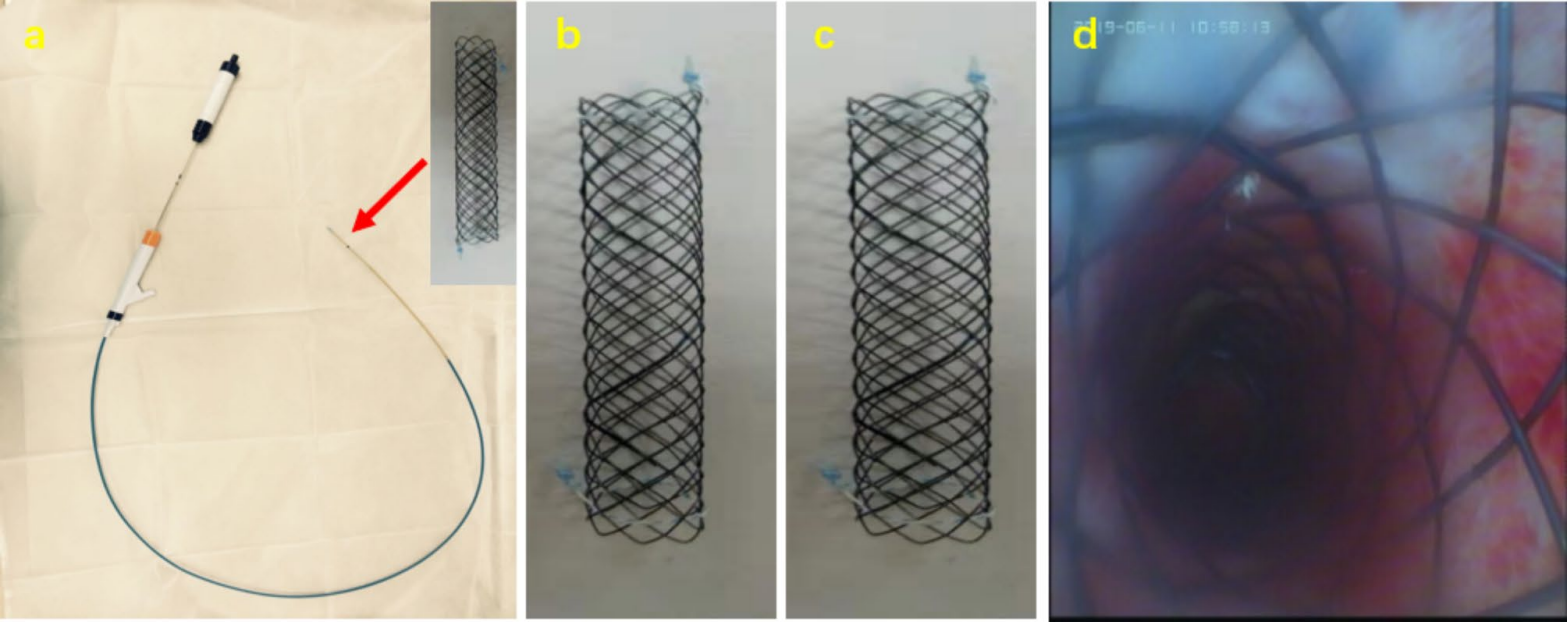
**a**: stent and delivery system; **b**: 6 mm*30mm airway stents; **c**: 8 mm*30mm airway stents; **d**: stent placement

The airway uncovered stent was self-expanding nickel-titanium alloy stent (Nanjing Micro-tech., Ltd., China) (Fig. 2a,b,c) with 30 mm of length and 6 or 8 mm of diameter. Knitting method: Single nickel-titanium alloy wire was woven into mesh structure in clockwise or counterclockwise spiral mode.

### Stent placement

The experimental rabbit was anesthetized and fixed well, and a 7 mm endotracheal tube inserted into the glottis. then the bronchoscope was used to locate the lesion, the guiding wire was delivered from the working channel of the bronchoscope to the distal end of the lesion, the device was left in the airway when the bronchoscope withdrawing, then the stent was delivered to the lesion site (Fig. 2d) according to the stent releasing methods [12],.

### Follow-up by bronchscopy and collection of pathological specimens

Intratracheal granulation hyperplasia and sputum retention were observed by bronchoscopy at 2nd week, 4th week, 8th week, and 12th week of stenting in Group A and B rabbits, Five rabbits was killed every two weeks till the end of 12 weeks of follow-up, 10ml air was injected through the ear vein, and the whole trachea was removed after rabbits was death. (Table 1). The tracheal samples were cut longitudinally to observe the condition of the tracheal wall and the mucosa at both ends of the stent. Tracheal stent samples were fixed in 10% buffered formalin and some fresh tracheal specimens were placed in the − 80℃ freezer.

**Table 1:** Each group of the number of bronchoscopic follow-up and tracheal samples

	2nd week	4th week	8th week	12th week
A	5	5	5	6
B	5	5	5	9

### Histopathologic Analysis

Tracheal samples were fixed in 10% buffered formalin and processed with the standard histologic paraffin technique with staining by hematoxylin and eosin. Slides were observed with an optical microscope (Nikon, Eclipse Ni; Nikon, Japan) with a digital output (Nikon DS-Ri2 camera).

### ELISA analysis IL-8, MMP9 and IL-1RA concentrations

Three 40-mg tracheal samples were taken from narrrow airway model rabbits (0 week), 2nd week, 4 th week, 8th week, and 12th week of procedure in group A, B, and the supernatant was reserved after homogenization centrifugation. IL-8, MMP9 and IL-1RA concentrations in 100ul samples were measured by ELISA (RayBiotech, Inc. Norcross, GA), according to the drug-set introduction.

### Statistical analysis

Statistically was analyzed with SPSS 22. The chi-square test was used e to compare the qualitative data., with Pearson’s chi-square test for T ≥ 5 and the continuity correction test for 1 ≤ T ≤ 5. The chi-square test/exact probability method was used to compare qualitative data. The Wilcoxon rank-sum test or T-tests was used for ordered classification data. *P* < 0.05 was used as the criterion for statistical significance.

## Results

### Statistical analysis of stent-related complications

After stent implanting, during the whole follow-up observation, tracheomalacia [13] occured in 1 of 21 rabbits (4.8%) in group A, all 24 rabbits (100%) in group B, there was significant differences (*P* < 0.05). The incidence rate of scar contracture at both ends of stent in group B (11of 24 45.8%) was significantly higher than that in group A (2/21,9.5%), there was significant differences (*P* < 0.05). Secret retention was 12 of 21 rabbits (57.1%) in group A, and in group B was17of 24 (70.8%), granulation was 11 of 21rabbits (52.3%) in group A and in group B was 18 of 24 (75%), about stent migration don’t occue in both group. There was no statistically significant difference in the incidence of stent migration, secretion retention, and granulation between the two groups of experimental rabbits (Table 2).

**Table 2:** Comparison of complications in A and B groups during the total observation

Complication	A(n = 21)	B(n = 24)	*P*
Tracheomalacia	1	24	9.74 × 10 ^-10
Scar contracture	2	11	0.02
Secretion retention	12	17	0.37
Granulation hyperplasia	11	18	0.13

### Tipical bronchoscopic, anatomiacal and histopathologic findings in stent group

**Fig. 3:**
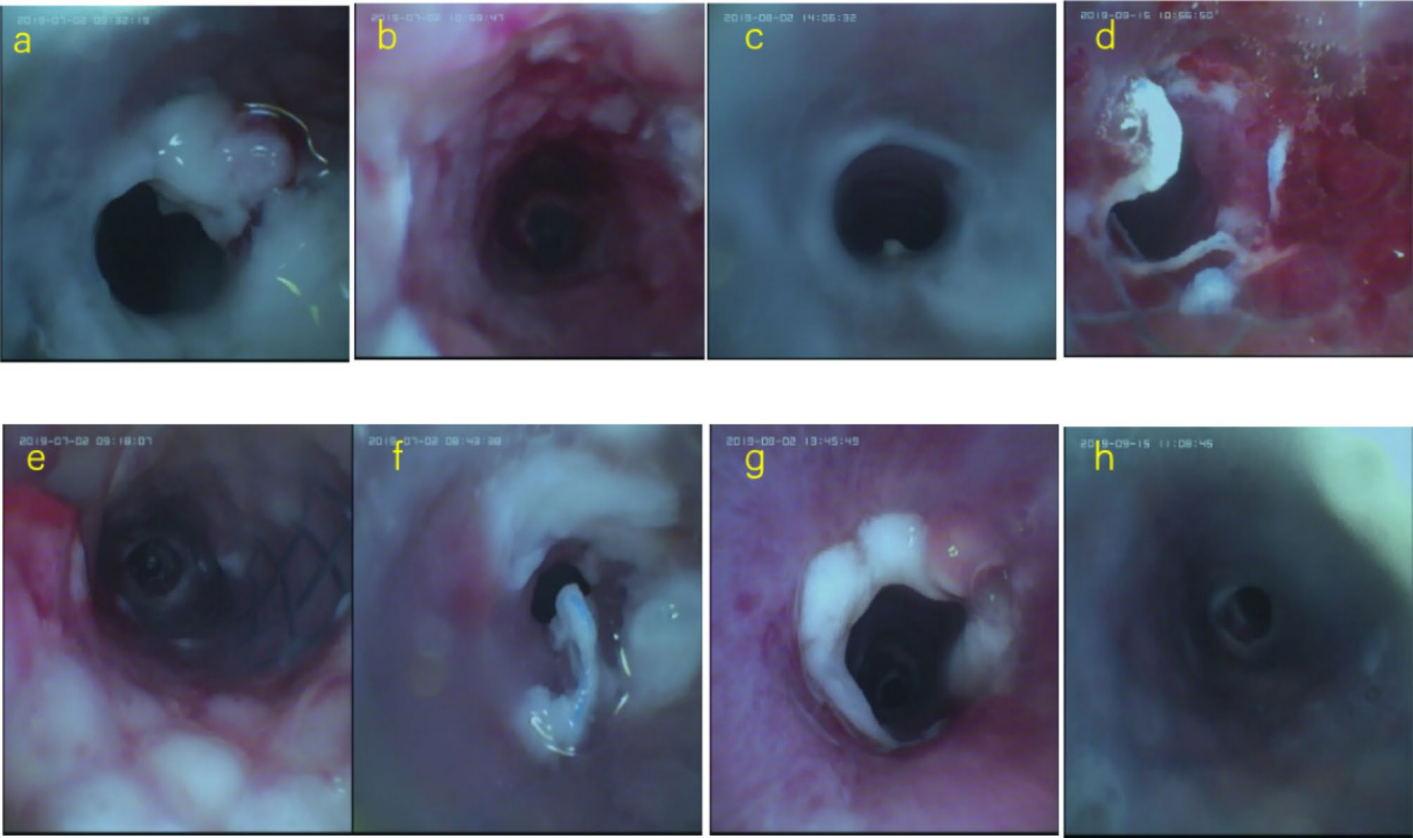
**a,e**: tracheal performance at the 2nd week after stenting in groups A and B, respectively; **b, f**: tracheal performance at the 4th-week in groups A and B, respectively; **c**, g:tracheal performance at the 8th-week in groups A and B, respectively; **d,h**: tracheal performance at the 12th-week in groups A and B, respectively

As for the complications of sputum retention and granulation, under bronchoscopy we observed that the complications occurred in rabbits after implantation 2 weeks, and gets worse before 8th week then alleviated (Fig. 3a, b, c, d and e), As for the complation of scar contracture and epithelization, we observed to begin in stenting 4th week and gets worse over time. (3f,g,h).

**Figs. 4:**
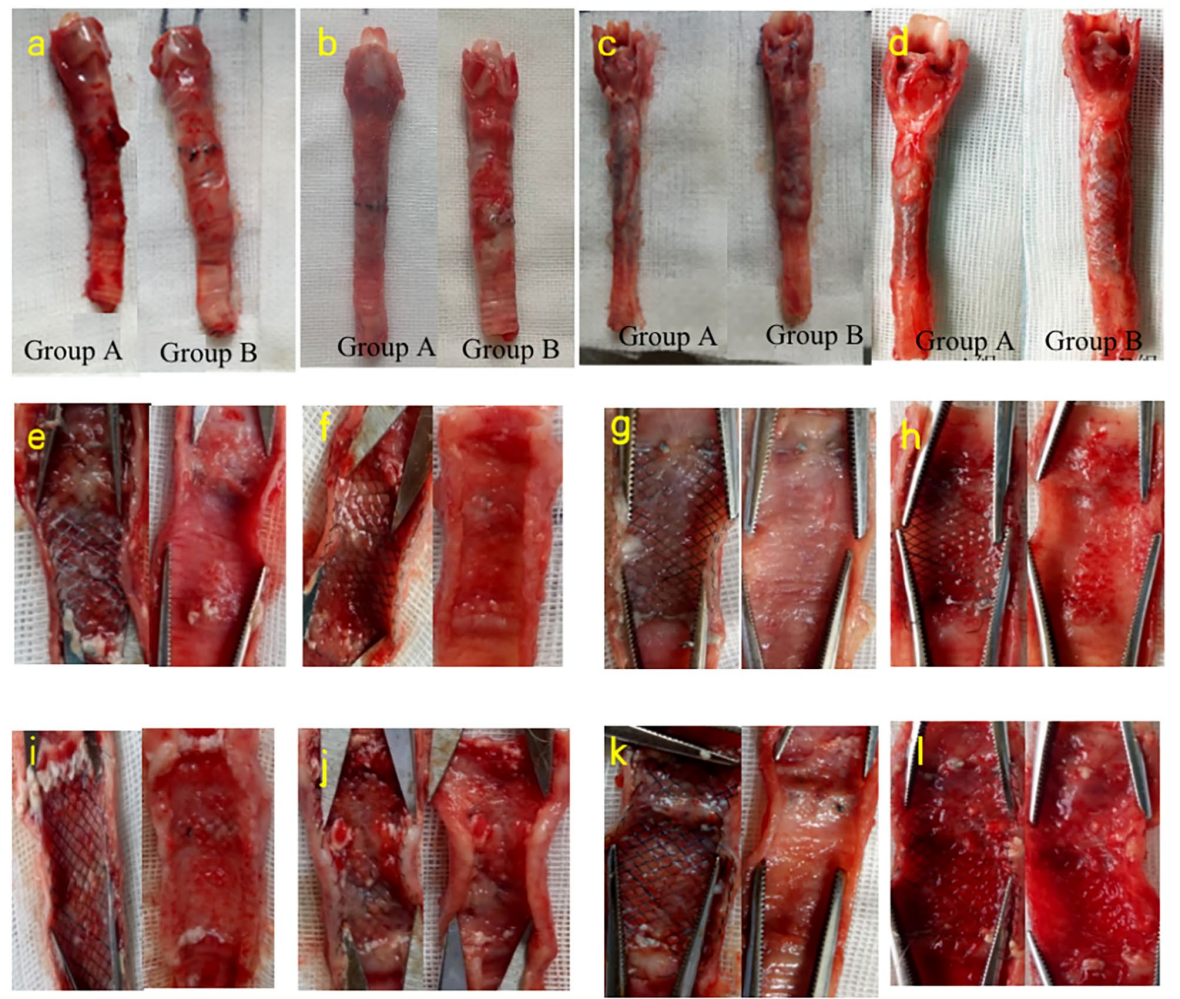
**a, b, c, d**: The gross anatomical maps at the 2nd, 4th, 8th and 12th week after stent implantation in groups A and B, respectively; **e,i**: the longitudinal maps at the 2nd week after stent implantation in groups A and B, respectively; **f, j**: the longitudinal maps at the 4th week after stent implantation in groups A and B, respectively; **g, k**: the longitudinal maps at the 8th week after stent implantation in groups A and B, respectively; **h, l**: the longitudinal maps at the 12th week after stent implantation in groups A and B, respectively

Five rabbits were sacrificed every 2 weeks, and we observed in gross anatomy, group B after stenting 2nd week, the trachal mucosa was congested and edematous, tracheomalacia appeared 2 weeks after implantation, and the scar contracture became worse at 8 weeks. In the 2nd week, the stent could be completely removed; In the 4th, 8th and 12th week, the stent could not be completely removed. (Fig. 4a-I).

**Fig. 5:**
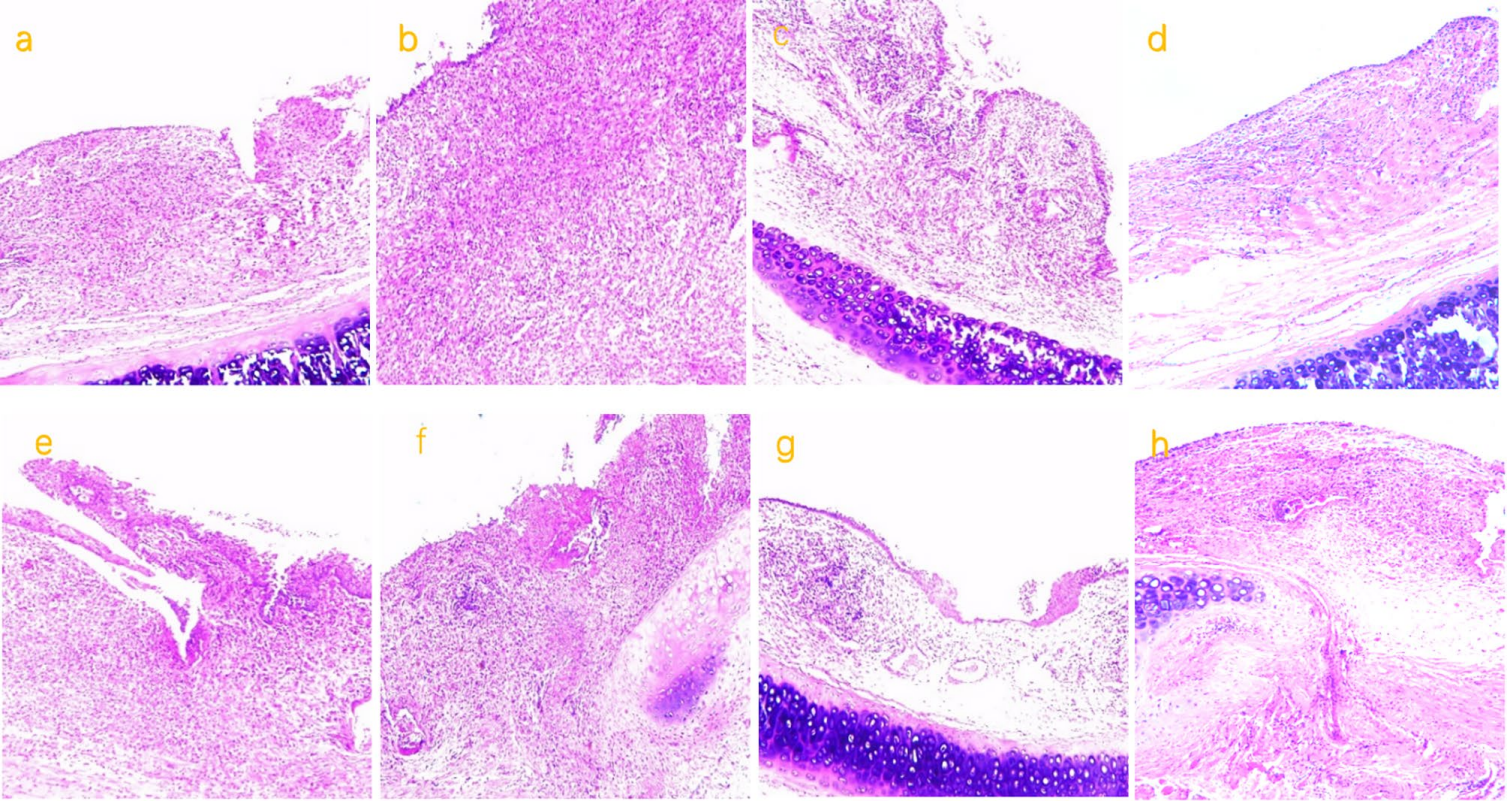
**a, e**: pathological images at 2nd week after stent implantation in experimental rabbits of groups A and B, respectively (X100); **b, f**: pathological images at 4th week after stent implantation in experimental rabbits of groups A and B, respectively (X100); **c, g**: pathological images at 8th week after stent implantation in experimental rabbits of groups A and B, respectively (X100); **d, h**: pathological images at 12th week after stent implantation in experimental rabbits of groups A and B, respectively (X100)

Histological findings are that the columnar epithelium of bronchial mucosa began to damage and detach, inflammatory cells infiltrated, mainly lobulated neutrophils from stenting 2th, after stenting 8th, we observed that the lamina propria glands almost disappeared, and collagen fiber significant proliferated, the tracheal epithelium was repaired. (Fig. 5a-h).

### Expression of IL-1RA, IL-8 and MMP9 in the stent group

The IL-1RA concentrations in the tracheal tissues of the stent groups at 2nd, 4th, 8th, and 12th week were 5.45 ± 1.12, 10.43 ± 4.98, 7.41 ± 4.69, and 5.23 ± 3.44 ng/mL, respectively, which were higher than those of the control group (2.32 ± 0.46 ng/mL), with the IL-1RA concentrations in the tracheal tissues of the stent groups at 2nd week and 4th week significantly higher than those of the control group, *P* < 0.05 (Table 3). The IL-8 concentrations in the tracheal tissue of the stent group at 2nd, 4th, 8th, and 12th week were 311.60 ± 8.13, 236.38 ± 57.22, 227.79 ± 21.83, and 212.42 ± 63.50 pg/mL, respectively, which were significantly higher than those of the control group (29.74 ± 6.44 pg/mL), *P* < 0.05 (Table 4), and the MMP9 concentrations in the tracheal tissue of the stent group at2nd, 4th, 8th, and 12th week were 990.26 ± 151.02, 698.91 ± 554.39, 1402.11 ± 961.00, and 495.54 ± 177.87 pg/mL, respectively, which were significantly higher than those of the control group (201.07 ± 179.23 pg/mL), *P* < 0.05 (Table 5).

**Table 3:** Comparison of IL-1RA concentrations between control and stent groups

	Control group	Stent group(n = 24)			
		2nd week	4th week	8th week	12th week
IL-1RA(ng/ml)	2.32 ± 0.46	5.45 ± 1.12	10.43 ± 4.98	7.41 ± 4.69	5.23 ± 3.44
*P*		6.53 × 10^-3	0.04	0.12	0.19

**Table 4:** Comparison of IL-8 concentrations between control and stent groups

	Control group	Stent group(n = 24)			
		2nd week	4th week	8th week	12th week
IL-8(pg/ml)	29.74 ± 6.44	311.60 ± 8.13	236.38 ± 57.22	227.79 ± 21.83	212.42 ± 63.50
*P*		6.56 × 10^-8	1.74 × 10^-3	1.24 × 10^-4	4.69 × 10^-3

**Table 5:** Comparison of MMP9 concentrations between control and stent groups

	Control group	Stent group(n = 24)			
		2nd week	4th week	8th week	12th week
MMP9(pg/ml)	201.07 ± 179.23	990.26 ± 151.02	698.91 ± 554.39	1402.11 ± 961.00	495.54 ± 177.87
*P*		1.44 × 10^-3	0.20	0.09	0.08

### Trend chart over time for IL-1RA, IL-8, MMP9 in stent group

**Fig. 6:**
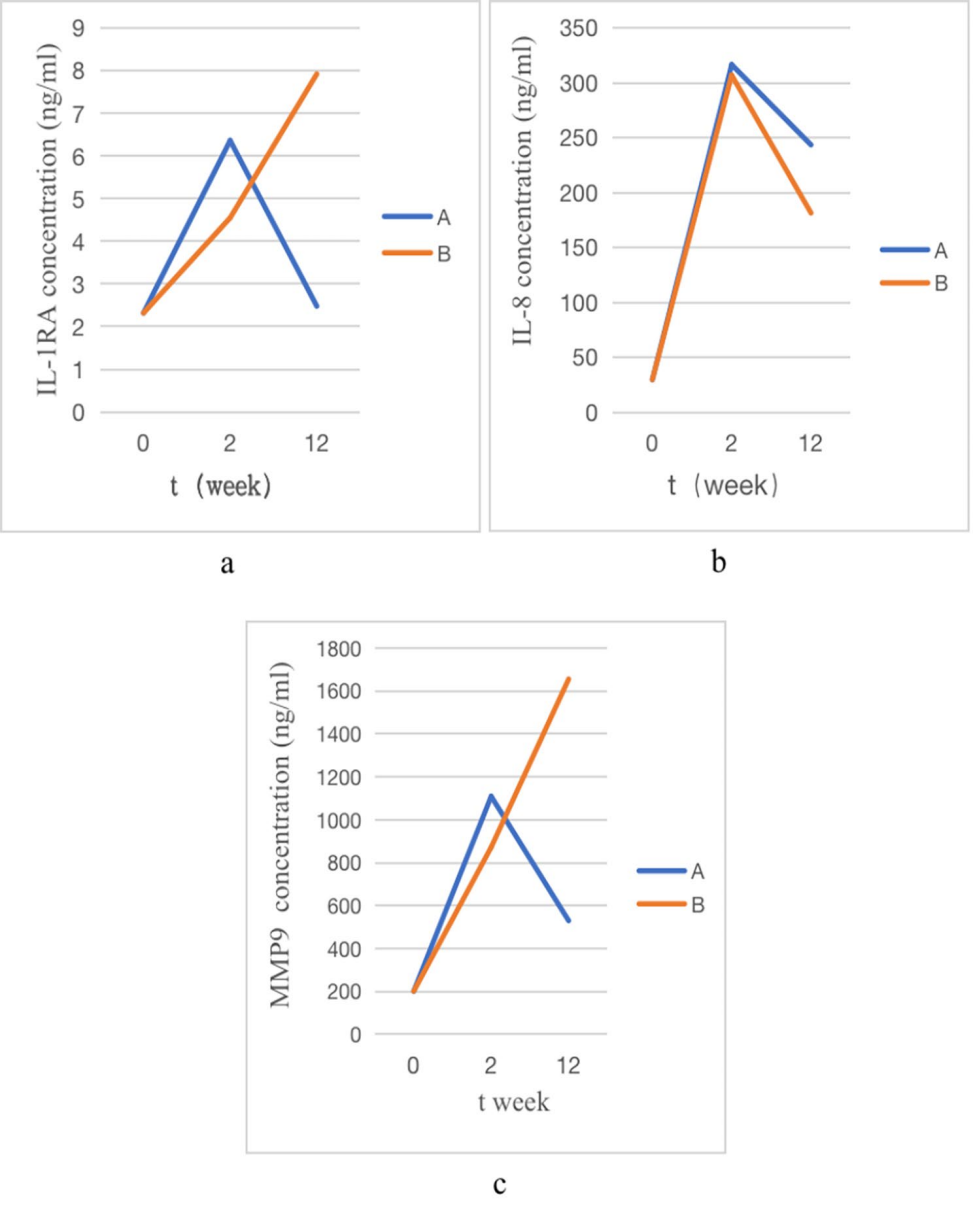
**a,b,c**: trend of IL-1RA, IL-8, MMP9 concentration in tracheal tissue at 0, 2nd and 12th week in groups A and B, respectively

After stent implantation in experimental rabbits, IL-1RA and MMP9 increased due to more severe inflammation in group B, while inflammatory factors gradually increased in the early stage of group A, but decreased after prolonged follow-up. IL-8 proinflammatory factors began to increase after stent implantation and showed a decreasing trend after prolonged follow-up, with no significant difference between groups A and B (Fig. 6a, b, and c).

## Discussion

Airways stents have been used to treat benign and malignant airway stenosis, SEM stent is limited in benign airway stenosis, Although SEM stent suggest in malignant airway stenosis, patients with advanced lung cancer also have focus on stent-related complacation because of their longer survival. [14, 15]. The optimal airway stent r can reduce the stent-related complacation. In China, metal stent are more widely used, but there are fewer studies on them. So we carried out a series of studies about China metal stent.

We chosed 4-month-old New Zealand white rabbits as experimental animals then established the tracheal stenosis by tracheotomy and scraping the mucosa with a brush, and destroying the cartilage with vascular forceps [11]. According to CT measurement of tracheal diameter and determine the airway stenosis rate, After a month, rabbit airway stenosis rate over 50% were put uncover metel stent. Group A stent is the ratio of stent diameter to airway diameter nearly 1.0 and group B stentsis the ratio of stent diameter to airway diameter over 1.2. Stent was made by Micro-Technology Company, China.

We compared two different diameters stent-related complications from bronchoscopic observation, gross anatomy, histopathology, and inflammatory factor (IL-1RA, IL-8 and MMP9). The main stent-related complications were granulation hyperplasia, scar contracture, stent migration, secretion retention, tracheomalacia after implantation every 2 week First, we selected the multiple inflammatory factors were detected the tracheal mucosa of rabbits after stent implantation by protein microarray, then the expression levels of IL-1RA, IL-8 and MMP9 were determined by ElISA to understand the mechanism of stent complications.

We observed granulation hyperplasia often occurred in the eyes of metal mesh and at both ends of the stent, and more significantly at both ends of the stent. In the early stage of stent implantation, granulation hyperplasia gradually increased, and in the 8th week, and 12th week granulation hyperplasia was alleviated From the 4th week, scar contracture at both ends of stent had formed and got woresned in group B and group A. During the whole process of follow-up, the incidence of scar contracture at both ends in group B was significantly higher than that in group A, *P* < 0.05.

After stent implantation, it will damage airway mucosal and then airway mucosal repair. The organism takes granulation hyperplasia and scar contracture ways to repair at different time, There can be divided into three sequential and overlapping processes, including inflammatory response, tissue repair and remodel after stent implation airways [16–18]. In addition, The expansion pressure of the stent and the stent- related infection and other factors can cause body to secrete a variety of cytokines to regulate the immune response [19]. Some studies suggest that it may be caused by the dysregulation of the interaction between pro-inflammatory response and anti-inflammatory response [20, 21]. Park [22]demonstrated that it could inhibit the formation of granulation tissue after urethral metal stent placement in rats by targeted inhibition of MMP9 expression. IL-8 as a proinflammatory factor, is involved in the acute and chronic inflammatory response after injury, and affects the function of fibroblasts, then promotes the proliferation and maturation of granulation tissue [23]. IL-1RA can competitively bind to IL-1 receptor and thus inhibit the inflammatory response, Nicolli [24]used a functional model of airway granulation tissue in laryngotracheal stenosis to intraperitoneally inject anti-IL-1RA into experimental mice to inhibit the role of IL-1 in the inflammatory cascade and prevent early granulation formation.

In our study, IL-8 in the stent group was significantly higher than that in the control group in the early stage, then showed a decreasing trend with the follow-up time, when IL-8 shows a decreasing trend, MMP9 increases and may continue to exert biological effects. IL-8 is a neutrophil chemotactic factor secreted by various cells and is crucial for inflammation and wound healing [25, 26, 27]. After tracheal wall injury, various cells release IL-8, participating in the inflammatory response and promoting the formation of granulation tissue [28]. Human IL-8 factor receptors include CXCR1 and CXCR2, when the concentration of IL-8 is low, CXCR1 receptor binds to it and thus secretes MMP9 to exert biological effects [29]. The concentration of MMP9 factor in the tracheal tissue of the stent group was significantly higher than that of the control group, MMP9 is a proteolytic enzyme family factor whose main function is to break down the extracellular matrix and participate in the activation of various cytokines, chemokines and growth factors [30], and is also a key mediator of tissue remodeling [31], which has an important role in maintaining physiological human tissue homeostasis [32]. Previous clinical studies on patients with uncover metal stents have shown that MMP9 antigen levels are associated with the development of restenosis [33, 34]. It has also been reported to analyze the expression level of MMP9 in human tissues after non-vascular stenting, confirming that MMP9 is a key target for stent-induced tissue proliferation [35]. MMP9 also has a variety of effects on lung diseases in clinical practice, and it has been reported that MMP9 is expressed as a mediator involved in injury-induced proliferation in normal human bronchial epithelial cells [36], and it is believed that MMP9 can accelerate the maturation of granulation tissue and the proliferation of fibroblasts [37]. Shin et al. [38] also confirmed that MMP9 was significantly overexpressed in stent-induced tracheal tissue proliferation and suggested that MMP9 may be a key prognostic factor for tissue proliferation after stent implantation.

The concentration of IL-1RA in the stent group was significantly higher than that in the control group, IL-1RA is a cytokine antagonist secreted by monocytes and macrophages that is able to competitively bind to the interleukin-1 receptor and does not trigger cells to produce any biological signal, thereby blocking the biological activity of IL-1 [39]. IL-1RA has already been demonstrated to play an important role in the process of restenosis after coronary stenting and is considered an important prognostic factor [40, 41]. Studies have shown that IL-1RA can inhibit the role of IL-1 in the inflammatory cascade to prevent early granulation formation in laryngotracheal stenotic airways, reflecting the important role IL-1RA plays in inhibiting granulation tissue formation and reducing inflammatory cell infiltration [24, 42, 43]., the role of IL-1RA in airway stents has not been studied. These results indicate that IL-IRA may be involved in the generation of stent complications.

Sum up, in our study, IL-8 began to rise and then fall trend after stent implation, which suggested involvement in the development of early complications. IL-RA and MMP9 showed a continuous upward trend in the large stent group, which may prolong or upregulate the inflammatory phase; In the small stent group IL-RA and MMP9 gradually increased in the early stage and decreased later as the tissue was gradually repaired. IL-1RA and MMP9 may be involved in the process of late proliferation and remodeling. Therefore, the diameter of metal stent is close to the normal airway diameter in the treatment of airway stenosis, which lead to the degree of inflammatory reaction relatively reduced, and the complications less than large-diameter tracheal stents. Uncover metal stents are generally used as temporary stent due to be difficulty to remove after implantation [44]. How long is easy to remove after stent implantation ?At present, there is no standard. In our study, it was difficult to remove the metal stent after the 4th weekWang et al. [45] also recommended that the uncover metal stent should be removed within one month.

. There are some limitations in this study that should be noted, First, The limitation of rabbit as an animal model is that the trachea diameter of rabbit is smaller than that of human, so the incidence of complications after stent implantation is higher than that of human, Nevertheless, our study aims to observe the difference of complications caused by stent diameter, so the results of the study are still helpful to clinical practice, Second, studies on the mechanism of complications after stent implantation are limited to a few inflammatory factors, which cannot fully explain causes of stent-related complications. The reason is that there are fewer antibodies to rabbit-derived inflammatory factors, We will increase the study of human specimens in the future.

## Conclusion

Implantation metal stents in rabbits with benign tracheal stenosis can produce varying degrees of stent-related complications, and small-diameter metal tracheal stents can reduce the incidence of tracheomalacia and scar contracture caused by stents. IL-1RA, IL-8 and MMP9 may be involved in the development of complications after stent implatation, Large-diameter metal tracheal stents caused peak backward of IL-1RA, IL-8 and MMP9.

## Data Availability

The data set supporting the results of this article are included within the article.

## Abbreviations

SEM: Self-expandable metallic
ELISA: Enzyme Linked Immunosorbent Assay
IL-1RA: Interleukin-1 receptor antagonist
CT: Computerized Tomography
IL-8: Interleukin-8
MMP9: Matrix metalloproteinase-9
SMZ: Compound Sulfamethoxazole Tablets
IL-1: Interleukin-1
